# 32 × 32 silicon electro-optic switch with built-in monitors and balanced-status units

**DOI:** 10.1038/srep42306

**Published:** 2017-02-09

**Authors:** Lei Qiao, Weijie Tang, Tao Chu

**Affiliations:** 1 State Key Laboratory on Integrated Optoelectronics, Institute of Semiconductors, Chinese Academy of Sciences, Beijing 100083, China; 2 College of Information Science and Electronic Engineering, Zhejiang University, Hangzhou, Zhejiang Province, 310027, China

## Abstract

To construct large-scale silicon electro-optical switches for optical interconnections, we developed a method using a limited number of power monitors inserted at certain positions to detect and determine the optimum operating points of all switch units to eliminate non-uniform effects arising from fabrication errors. We also introduced an optical phase bias to one phase-shifter arm of a Mach–Zehnder interferometer (MZI)-type switch unit to balance the two operation statuses of a silicon electro-optical switch during push–pull operation. With these methods, a 32 × 32 MZI-based silicon electro-optical switch was successfully fabricated with 180-nm complementary metal–oxide–semiconductor (CMOS) process technology, which is the largest scale silicon electro-optical switch to the best of our knowledge. At a wavelength of 1520 nm, the on-chip insertion losses were 12.9 to 16.5 dB, and the crosstalk ranged from −17.9 to −24.8 dB when all units were set to the ‘Cross’ status. The losses were 14.4 to 18.5 dB, and the crosstalk ranged from −15.1 to −19.0 dB when all units were in the ‘Bar’ status. The total power consumptions of the 32 × 32 switch were 247.4 and 542.3 mW when all units were set to the ‘Cross’ and ‘Bar’ statuses, respectively.

Accompanying the rapid increases in the numbers of data centres (DCs) and high-performance computers (HPCs), optical switches are highly demanded for the construction of DC and HPC optical interconnection networks for increasing the speed of data exchange and reducing power consumption^1^,^2^,^3^. These switches are also key devices for building all-optical telecommunication networks. Silicon-integrated optical switches, together with other silicon photonic devices based on silicon-on-insulator (SOI) substrates and complementary metal–oxide–semiconductor (CMOS)-compatible process technologies, are widely regarded as the most promising devices for applications in data communications and telecommunications. They are compact, have a low power consumption and low cost, and are fit for large-scale monolithic integration.

Many studies of silicon switches have been conducted^4^,^5^,^6^,^7^,^8^,^9^,^10^,^11^,^12^,^13^. Compared to a thermal-optical (TO) switch, a silicon electro-optical (EO) switch with a nanosecond switching speed is more desirable since it can switch sufficiently fast to work as an optical packet switch in data communications, especially in HPCs, as well as in telecommunications. The silicon switches that have been previously reported are usually based on Benes, Spanke–Benes, crossbar, and tree network structures^14^,^15^,^16^,^17^. Among them, the Benes network is a re-arrangeable non-blocking network with fewer switch units, resulting in a lower insertion loss and larger scale integration.

Nevertheless, in contrast to their compact footprint, silicon switches are sensitive to unexpected fabrication variations^18^. That is, submicron section-size variations in silicon waveguides change the effective refractive indices and the operating points of the switch units. This results in a severe difficulty in realising large-scale silicon switches since it is difficult to operate each switch unit at its optimum points with less crosstalk in a large-scale N × N switch. Another critical issue in an EO Mach–Zehnder interferometer (MZI) switch whose bandwidth and stability are much better than a micro-ring switch, is the performance difference arising from the absorption of injected free carriers in the switch’s ‘Cross’ and ‘Bar’ statuses. The insertion loss and crosstalk of switches that are all in the ‘Bar’ status are greatly deteriorated, especially in a large-scale switch^12^.

In this paper, we propose a novel method to detect and determine the optimum operating points of a Benes-network-based switch and all of its switch units with a limited number of built-in power monitors. This method is more effective for achieving reductions in both the chip size and measurement time than the addition of power monitors to every switch unit^12^,^19^. We also propose a method for setting the optical phase bias in the MZI phase-shift arm to eliminate the difference between the switch’s ‘Cross’ and ‘Bar’ statuses with push–pull operation control and no extra heat power added for phase tuning. With these methods, we experimentally demonstrated a 32 × 32 MZI-based silicon EO switch fabricated with 180-nm CMOS process technology. To the best of our knowledge, this is the largest scale silicon EO switch worldwide. At a wavelength of 1520 nm, the on-chip insertion losses were from 12.9 to 16.5 dB, and the crosstalk ranged from −17.9 to −24.8 dB when all units were in the ‘Cross’ status with a power consumption of 247.4 mW. Moreover, the on-chip insertion losses were from 14.4 to 18.5 dB, and the crosstalk ranged from −15.1 to −19.0 dB when all units were in the ‘Bar’ status with a power consumption of 542.3 mW.

## Results

### Monitoring switch of a Benes network

**Figure 1:**
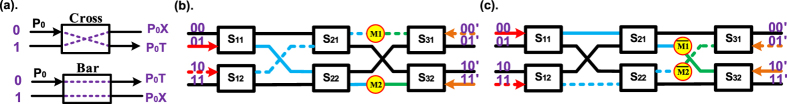
Statuses of a switch unit and detection using various ports. (**a**) Two statuses of a switch unit: Cross and Bar. (**b**) Detection using left ports 01 and 10 and right ports 00′ and 11′ as input ports. (**c**) Detection using left ports 00 and 11 and right ports 01′ and 10′ as input ports.

A Benes network is an iterative network based on 2 × 2 and 4 × 4 basic units, which are helpful for finding a common rule for inserting limited numbers of monitors at defined positions. A crosstalk analysis of a Benes switch can be easily understood starting with 2 × 2 and 4 × 4 switches. Ignoring the propagating loss of the switch units, the crosstalk of a 2 × 2 switch can be defined as X = (P_in_ − P_out_)/P_in_ = 1 − T, where the transmittance T is the ratio of the output power P_out_ of the selected port to the input power P_in_, as shown in Fig. 1(a). The operation point of a 2 × 2 switch unit can be determined and adjusted by detecting its minimal crosstalk power, which is more sensitive to the operation status than the transmission.

The model of light propagation for a 4 × 4 switch can be defined as shown in Fig. 1(b), where S_ij_ stands for the switch unit in stage i and line j, and the input port of a switch is labelled with a binary number, as described elsewhere^20^. X_ij_ and T_ij_ are the crosstalk and transmission of unit S_ij_, respectively. The crosstalk and insertion loss of waveguide crossings were ignored for a simpler analysis^21^,^22^. Assuming that all of the switch units are working in the ‘Cross’ status and light is input from left-side port 01 with a power of P_0_, the output power of each switch unit is listed in Table 1.

In Table 1, the crosstalk X_11_ of S_11_ can be detected by measuring the outputs of the 1^st^ stage, P_0_X_11_, and 2^nd^ stage, P_0_X_11_T_22_. These outputs have a simple proportional relationship with the crosstalk X_11_ without being affected by the switch’s extinction, which allows for the minimum value of X_11_ to be measured easily. The power monitor could be a contactless optical probe or tuneable coupler^23^,^24^, which has less influence on the switch’s performance. As shown in Fig. 1(b), the crosstalk of S_11_ and S_21_ could be obtained by detecting the output of the 2^nd^ stage, P_0_X_11_T_22_ and P_0_T_11_X_21_, by monitors M2 and M1 when light is input from left port 01. However, the 3^rd^ stage output is the interference result of the main power and crosstalk power. The crosstalk is difficult to detect by measuring this output, even when the maximum output power in Table 1 is considered. By changing the input port to 10, the crosstalk of S_12_ and S_22_ can also be detected with monitors M1 and M2. The units in the 3^rd^ stage can be directly detected by the same monitors, M2 and M1, with the light input from the right-side port of the switch. The crosstalk of all switch units can also be measured when all units are set to the ‘Bar’ status, as summarised in Table 2. Therefore, only two monitors, M1 and M2, are needed to detect the operations of each switch unit in a 4 × 4 Benes network. Another detection method with different inputs (00 and 11) and power monitors (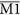 and 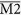) is illustrated in Fig. 1(c). In this method, the monitors are called complementary monitors compared to those in previous method. With these complementary monitors, light can be input from all input ports of a 4 × 4 Benes network for detection.

### 8 × 8 monitoring experiment

**Figure 2:**
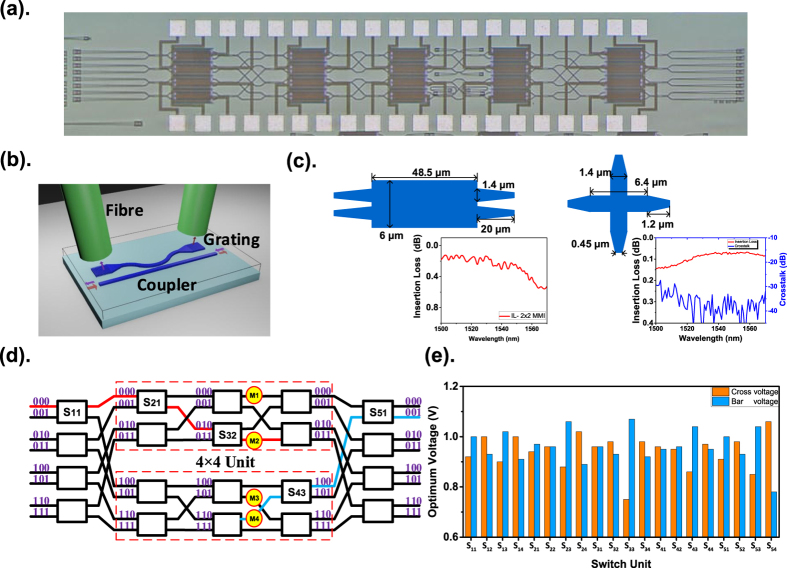
Detection schematic and results for the units in the 8 × 8 switch. (**a**) Micrograph of an 8 × 8 Benes switch fabricated with 180-nm CMOS process technology. (**b**) Bi-directional couplers used as power monitors, which can detect bi-directional light power in the waveguide. (**c**) Network of an 8 × 8 Benes switch consisting of two 4 × 4 Benes switches embedded with power monitors. (**d**) The optimum operation voltage of the switch units in an 8 × 8 switch. (**e**) The design dimensions and test results for a 2 × 2 MMI and waveguide crossing.

An 8 × 8 Benes switch is fabricated with 180-nm CMOS process technology to validate the detection method. In Fig. 2(a), the 8 × 8 switch has 20 switch units and four 10% bi-directional power taps used as monitors. The bi-directional coupler in Fig. 2(b) consists of a directional coupler and two grating fibre couplers and detects the light in the waveguide from both the left and right sides. The bi-directional coupler had a 250-nm-wide gap between a straight waveguide and a bent waveguide with a 20-μm bend radius. The grating coupler was designed with a 610-nm period and 60% duty, which has a −5 dB coupling efficiency at 1550 nm with a 10° fibre angle. The switch is built up by multimode interferometers (MMIs) and waveguide crossings. The dimensions and test result are shown in Fig. 2(c). The insertion loss of the MMI is from 0.12 to 0.56 dB from 1500 to 1570 nm, which was obtained by a linear fitting of the measured losses of the cascade MMIs. The crosstalk of the waveguide crossing is less than −30 dB, and the insertion loss is from 0.07 to 0.15 dB from 1515 to 1570 nm.

Figure 2(d) shows an 8 × 8 Benes switch consisting of two 4 × 4 Benes switches embedded with power monitors in complementary positions. In a Benes network, the transmission light power and crosstalk power of the 1^st^ stage unit propagate to the upper and lower 4 × 4 switches, respectively. Taking the output of the 1^st^ stage in the 8 × 8 switch as the 4 × 4 switch input, the first two stages of the 4 × 4 switch units can be detected with the methods described before. The crosstalk light in the 1^st^ stage passes through the first two stages of a 4 × 4 switch and can also be detected by the monitors. For example, by setting all of the switch units to the ‘Cross’ status with light input from left-side port 000, the crosstalk light of S_11_ passing through the path S_11_ → S_21_ → S_32_ → M2 is detected by monitor M2, as indicated by the red line in Fig. 2(d). The units on the right side of the monitors can be detected with the incident light input from the right-side ports. For example, for incident light from right-side port 001’, the crosstalk power of S_51_ propagates through the route S_51_ → S_43_ → M4, as indicated by the blue line. Similarly, all other units can be detected by four monitors and adjusted to their optimum operating points according to the method in Table 3.

Figure 2(e) shows the optimum operation voltage measured for each unit of the 8 × 8 switch. After adjustment with these results, the crosstalk of the 8 × 8 switch was measured for each input port at 1550 nm when all units were in the ‘Cross’ and ‘Bar’ statuses. The crosstalk ranges from −21.3 to −16.6 dB in the ‘Cross’ status and from −24.3 to −13.5 dB in the ‘Bar’ status.

### N × N switch evolution

**Figure 3:**
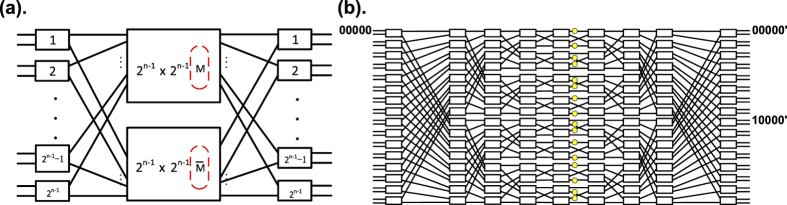
Universal monitor detection method for a 2^*n*^ × 2^*n*^ Benes network. (**a**) The monitor positions in the two 2^*n*−1^ × 2^*n*−1^ subnetworks are complementary to each other, and the crosstalk of all units can be monitored with these monitors. (**b**) Network structure of a 32 × 32 switch with 16 built-in power monitors set by the universal method.

The applicability of the monitoring method can be extended with induction to a 2^*n*^ × 2^*n*^ Benes network switch as a universal method, which is shown in Fig. 3(a). For a 2^*n*^ × 2^*n*^ network, the monitor positions in the upper 2^*n−*1^ × 2^*n−*1^ Benes network are complementary to the lower 2^*n−*1^ × 2^*n−*1^ Benes network. Since this method is applicable to 4 × 4 and 8 × 8 Benes switches, we may assume that the method is also applicable to a 2^*n*−1^ × 2^*n−*1^ Benes switch. Light passes through a switch unit in the input stage and is input into two 2^*n−*1^ × 2^*n*−1^ subnetworks; the inputs of the 2^*n−*1^ × 2^*n−*1^ networks contain the status information of the unit in the input stage. Since the monitor positions in these two 2^*n*−1^ × 2^*n*−1^ networks are complementary to each other, the crosstalk power of the unit in the 1^st^ stage can always be monitored by the monitor in the upper or lower 2^*n*−1^ × 2^*n−*1^ networks. All units in the input and output stages of the 2^*n*^ × 2^*n*^ network can be detected by the monitors in two 2^*n−*1^ × 2^*n−*1^ networks. Thus, we can conclude that the monitoring method is applicable to a 2^*n*^ × 2^*n*^ Benes network switch.

### Optical Phase Bias

**Figure 4:**
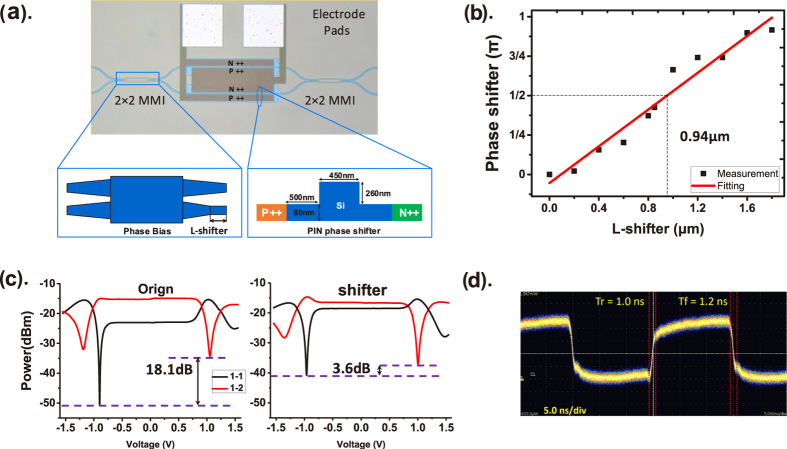
Schematic and performance of the optical phase bias. (**a**) Switch unit, the MMI with the optical phase bias structure, and the cross section of the PIN phase shifter. (**b**) Relation between the phase shift and the length of the MMI shifter. (**c**) Improvement in the crosstalk by a phase bias. (**d**) Switching time of the switch unit with a phase bias.

Usually, an MZI switch has equally long phase-shift arms. It needs a phase shift of π to switch between the ‘Cross’ and ‘Bar’ statuses. The switch we designed has two 200-μm-long PIN phase-shift arms with the cross section shown in Fig. 4(a). Phase shifting in a EO switch uses the plasma dispersion effect caused by the injected carriers, which also causes optical absorption. The optical absorption loss introduces an imbalance in the two arms and increases crosstalk. Here, we propose a method that sets a phase bias of π/2 in one MZI arm and uses push–pull operation to decrease the phase shift needed for switching by changing the waveguide structure. A lower phase shift results in less optical loss and less crosstalk. As shown in Fig. 4(a), the phase bias is provided by decreasing the length of the lower output taper in the left MMI and filling with a straight waveguide called an L-shifter. The optimised bias length of the L-shifter of 0.9 μm was obtained by measuring the ratio of the two outputs, as shown in Fig. 4(b). A comparison of a phase-bias switch and an origin unit without a bias is shown in Fig. 4(c). The difference in the crosstalk between the ‘Cross’ and ‘Bar’ was improved from 18.1 to 3.6 dB. The power consumption was also reduced from 6.24 to 1.9 mW in the ‘Bar’ status. The switching time of the switch unit was measured with the falling and rising times of 1.0 and 1.2 ns, respectively, as shown in Fig. 4(d).

### 32 × 32 switch experiment

**Figure 5:**
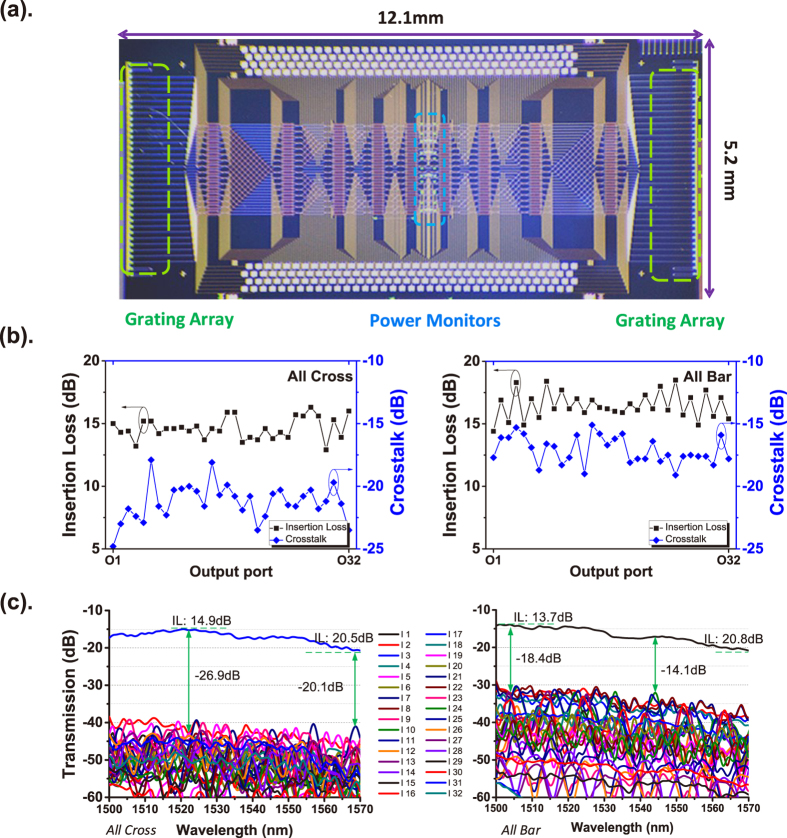
Micrograph and optical performance of a 32 × 32 switch. (**a**) Micrograph. (**b**) Crosstalk and on-chip insertion loss results for all output ports when all units were in the ‘Cross’ and ‘Bar’ statuses at a wavelength of 1520 nm. (**c**) Crosstalk and on-chip insertion loss (IL) spectra for output port 00000’ of all units in the ‘Cross’ and ‘Bar’ statuses.

With the methods proposed above, the 32 × 32 silicon EO switch with dimensions of 12.1 mm × 5.2 mm in Fig. 5(a) was fabricated. It has 144 phase-bias 2 × 2 MZI-switch units and 288 electrode pads; 16 bi-directional power taps were implemented in the switch to detect the operation statuses of all 144 units, as shown in Fig. 3(b). First, the operation points for all units in the ‘Cross’ and ‘Bar’ statuses were investigated with the 16 power taps. The total power consumption of the 32 × 32 switch was measured to be 247.4 and 542.3 mW when all units were in the ‘Cross’ and Bar’ statuses, respectively. More details are presented in Supplementary Tables S1 and S2. Since the 32 × 32 switch has 32! = 2.63 × 10^35^ routing states, it is very time-consuming to test all of them. The crosstalk and on-chip insertion losses of all output ports were measured when all units were in the ‘Cross’ and ‘Bar’ statuses at a wavelength of 1520 nm, and more details are presented in Supplementary Figs S1 and S2. The results are shown in Fig. 5(b). The on-chip insertion losses were 12.9 to 16.5 dB, and the crosstalk ranged from −17.9 to −24.8 dB when all units were in the ‘Cross’ status. The on-chip insertion losses were 14.4 to 18.5 dB, and the crosstalk ranged from −15.1 to −19.0 dB when all units were in the ‘Bar’ status. Since the Benes network has same number of switch units and a connection waveguide with almost the same length, the total insertion loss of the switch is mainly influenced by the number of crossings in the path. The insertion loss of a waveguide crossing is 0.11 dB at 1520 nm. On the basis of the results for the insertion losses of the paths in the ‘Cross’ and ‘Bar’ statuses at 1520 nm, the average losses of each stage (switch unit and connection waveguide) excluding the crossings were 1.30 dB per stage in the ‘Cross’ status and 1.50 dB in the ‘Bar’ status. The loss of every path could be budgeted by adding its crossing loss and stage loss. The bandwidth performance of output port 00000’ was tested as a representative situation, as shown in Fig. 5(c), and all units which were passed through were in the ‘Cross’ and ‘Bar’ statuses. For wavelengths in the range of 1500–1570 nm, the crosstalk was measured to be −26.9 to −20.1 dB, and the on-chip insertion losses were 14.9 to 20.5 dB when all units were in the ‘Cross’ status. Further, the crosstalk was −18.4 to −14.1 dB, and the on-chip insertion losses were 13.7 to 20.8 dB when all units were in the ‘Bar’ status.

## Discussion

We have demonstrated a method using limited numbers of power monitors inserted at certain positions to detect and determine the optimum operating points of all switch units in a large-scale silicon optical switch. This method more effectively realises reductions in both the chip size and measurement cost compared to the addition of power monitors to each switch unit. We also introduced an optical phase bias to one arm of an MZI to balance the performance of two operation statuses of a silicon EO switch by push–pull operation. With these methods, a 32 × 32 MZI-based EO switch was successfully demonstrated on a 180-nm CMOS process platform, which is the largest scale silicon EO switch to the best of our knowledge. The total power consumptions of the 32 × 32 switch were 247.4 and 542.3 mW when all units were in the ‘Cross’ and ‘Bar’ statuses, respectively. At a wavelength of 1520 nm, the on-chip insertion losses were 12.9 to 16.5 dB, and the crosstalk ranged from −17.9 to −24.8 dB when all units were in the ‘Cross’ status. The on-chip insertion losses were 14.4 to 18.5 dB, and the crosstalk ranged from −15.1 to −19.0 dB when all units were in the ‘Bar’ status. In the 70-nm bandwidth of 1500–1570 nm, the crosstalk of output port 00000’ was −26.9 to −20.1 dB, and the on-chip insertion losses were 14.9 to 20.5 dB when all units were in the ‘Cross’ status. In contrast, the crosstalk was −18.4 to −14.1 dB, and the on-chip insertion losses were 13.7 to 20.8 dB when all units were in the ‘Bar’ status. The worst on-chip insertion losses could be estimated depending on the bandwidth performance of the passive components^13^. The worst on-chip insertion losses in the all ‘Cross’ and all ‘Bar’ statuses were calculated to be 22.6 and 24.3 dB, respectively. The worst crosstalk would deteriorate as the wavelength increases because of the change in the split ratio of the MMI at a longer wavelength. In future studies, although the uniformity of the switch units can be improved by using a finer process such as 90-nm and 45-nm CMOS technology, a monitoring method is still needed since the several-nanometre variation in the waveguide width might cause a phase change of π/2 in a 200-μm-long phase shifter. On the other hand, although the loss of a switch unit might be improved to about 1 dB, built-in amplifiers would be needed to build larger silicon EO switches for reducing the insertion loss of the switch. The polarization dependence of the silicon optical switch using a submicron waveguide is also a significant issue for use in practical optical communication. Polarization control devices^25^ and polarization-independent silicon waveguides^26^ could be used to solve this problem.

## Methods

### Fabrication

All of the silicon optical switches mentioned in this paper were fabricated with the 180-nm CMOS process line of Semiconductor Manufacturing International Corporation (SMIC) on the basis of a 340 nm silicon-on-insulator (SOI) platform. The connecting rib waveguide has a width of 450 nm with an 80-nm-high slab, which has an average propagation loss of 0.53 dB/mm at 1550 nm wavelength. All of the passive components such as the MMIs, waveguide crossings, and grating couplers were designed for the TE mode. The P++ and N++ doping densities are approximately 2 × 10^20^ cm^−3^. The metal wires are 0.45-μm-thick aluminium wires. Trenches with an area of 240 μm × 4.5 μm were inserted between adjacent PIN phase shifters to avoid thermal and electrical crosstalk.

### Measurement

The crosstalk of one port of an optical switch is defined as the ratio between the output power from the target input port and that from all other ports when light was launched into all input ports. The on-chip insertion loss excluded the grating coupling loss spectrum. The switch chip mounted on the heat sink was connected to the test board using wire bonding. A tuneable laser (SANTEC TSL-510), an optical power meter (YOKOGAWA AQ2200), an optical spectrum analyser (YOKOGAWA AQ6370C), a high-speed sampling oscilloscope (Tektronix DSA 8300), a pulse pattern generator (Agilent 81130 A), and direct-current power supplies (Agilent E3631A and B2901A) were used to test the static and dynamic characters of the switches.

## Additional Information

**How to cite this article**: Qiao, L. *et al*. 32 × 32 silicon electro-optic switch with built-in monitors and balanced-status units. *Sci. Rep.* **7**, 42306; doi: 10.1038/srep42306 (2017).

**Publisher's note:** Springer Nature remains neutral with regard to jurisdictional claims in published maps and institutional affiliations.

## Supplementary Material

Supplementary Information

## Figures and Tables

**Table 1:** Output power of each stage in a 4 × 4 switch in the ‘Cross’ status.

Input (Port)	Stage 1	Stage 2	Stage 3 (Max)
0 (00)	P_0_T_11_	P_0_T_11_X_21_	P_0_T_11_X_21_X_31_ + P_0_X_11_X_22_T_31_
P_0_ (01)	P_0_X_11_	P_0_T_11_T_21_	P_0_T_11_X_21_T_31_ + P_0_X_11_X_22_X_31_
0 (10)	0	P_0_X_11_X_22_	P_0_T_11_T_21_X_32_ + P_0_X_11_T_22_T_32_
0 (11)	0	P_0_X_11_T_22_	P_0_T_11_T_21_T_32_ + P_0_X_11_T_22_X_32_

**Table 2:** Detection method for the units of a 4 × 4 switch using M1 and M2.

Switch status	In port, left side	M1 power	M2 power	Detected unit	In port, right side	M1 power	M2 power	Detected unit
All ‘Cross’	01	P_0_T_11_X_21_	P_0_X_11_T_22_	S_11_, S_21_	00’	P_0_X_31_	—	S_31_
	10	P_0_X_12_T_21_	P_0_T_12_X_22_	S_12_, S_22_	11’	—	P_0_X_32_	S_32_
All ‘Bar’	01	P_0_X_11_T_21_	P_0_T_11_X_22_	S_11_, S_22_	01’	P_0_X_31_	—	S_31_
	10	P_0_T_12_X_21_	P_0_X_12_T_22_	S_12_, S_21_	10’	—	P_0_X_32_	S_32_

**Table 3:** Detection method for the units of an 8 × 8 switch.

Port in	Switch	Monitor	Switch	Monitor	Switch	Monitor	Port in	Switch	Monitor	Switch	Monitor
**All ‘Cross’**											
000	S_11_	M2	S_23_	M3	S_34_	M4	001’	S_51_	M4	S_41_	M1
011	S_12_	M3	S_21_	M2	S_31_	M1	010’	S_52_	M1	S_43_	M4
101	S_13_	M4	S_22_	M1	S_32_	M2	100’	S_53_	M2	S_44_	M3
110	S_14_	M1	S_24_	M4	S_33_	M3	111’	S_54_	M3	S_42_	M2
**All ‘Bar’**											
001	S_11_	M1	S_23_	M4	S_33_	M3	001’	S_51_	M1	S_43_	M4
010	S_12_	M4	S_21_	M1	S_32_	M2	010’	S_52_	M4	S_41_	M1
100	S_13_	M3	S_22_	M2	S_31_	M1	100’	S_53_	M3	S_42_	M2
111	S_14_	M2	S_24_	M3	S_34_	M4	111’	S_54_	M2	S_44_	M3

## References

[b1] Soref R. The past, present, and future of silicon photonics. Selected Topics in Quantum Electronics, IEEE Journal of 12, 1678–1687 (2006).

[b2] Rumley S. . Silicon photonics for exascale systems. Journal of Lightwave Technology 33, 547–562 (2015).

[b3] Nikolova D. . Scaling silicon photonic switch fabrics for data center interconnection networks. Optics express 23, 1159–1175 (2015) 25835876 10.1364/OE.23.001159

[b4] Chu T. . Compact 1 × n thermo-optic switches based on silicon photonic wire waveguides. Optics Express 13, 10109–10114 (2005). 19503224 10.1364/opex.13.010109

[b5] Yang M. . Non-blocking 4 × 4 electro-optic silicon switch for on-chip photonic networks. Optics express 19, 47–54 (2011). 21263541 10.1364/OE.19.000047

[b6] Xing J. . Nonblocking 4 × 4 silicon electro-optic switch matrix with push–pull drive. Optics letters 38, 3926–3929 (2013). 24081090 10.1364/OL.38.003926

[b7] Chen L. . Compact, low-loss and low-power 8 × 8 broadband silicon optical switch. Optics express 20, 18977–18985 (2012). 23038537 10.1364/OE.20.018977

[b8] Tanizawa K. . Ultra-compact 32 × 32 strictly-non-blocking Si-wire optical switch with fan-out LGA interposer. Optics express 23, 17599–17606 (2015). 26191767 10.1364/OE.23.017599

[b9] Lee B. . Monolithic silicon integration of scaled photonic switch fabrics, cmos logic, and device driver circuits. Lightwave Technology, Journal of 32, 743–751 (2014).

[b10] Nakamura S. . Compact and low-loss 8x8 silicon photonic switch module for transponder aggregators in cdc-roadm application. In Optical Fiber Communication Conference, M2B–6 (Optical Society of America, 2015).

[b11] Qiao L. . 16 × 16 non-blocking silicon electro-optic switch based on Mach-Zehnder interferometers. In Optical Fiber Communication Conference, Th1C–2 (Optical Society of America, 2016).

[b12] Lu L. . 16 × 16 non-blocking silicon optical switch based on electro-optic Mach-Zehnder interferometers. Optics express 24, 9295–9307 (2016). 27137545 10.1364/OE.24.009295

[b13] Seok T. J. . Large-scale broadband digital silicon photonic switches with vertical adiabatic couplers. Optica 3, 64–70 (2016)

[b14] Benes V. Algebraic and topological properties of connecting networks. Bell System Technical Journal 41, 1249–1274 (1962).

[b15] Spanke R. . N-stage planar optical permutation network. Applied Optics 26, 1226–1229 (1987). 20454306 10.1364/AO.26.001226

[b16] Nishi T. . A polarization-controlled free-space photonic switch based on a pi-loss switch. Photonics Technology Letters, IEEE 5, 1104–1106 (1993).

[b17] Spanke R. A. Architectures for large nonblocking optical space switches. Quantum Electronics, IEEE Journal of 22, 964–967 (1986).

[b18] Selvaraja S. K. . Subnanometer linewidth uniformity in silicon nanophotonic waveguide devices using cmos fabrication technology. Selected Topics in Quantum Electronics, IEEE Journal of 16, 316–324 (2010).

[b19] Morichetti F. . Contactless integrated photonic probe: Concept, technology and applications. In Optical Fiber Communication Conference, M2I–1 (Optical Society of America, 2016).

[b20] Lawrie D. H. Access and alignment of data in an array processor. Computers, IEEE Transactions on 100, 1145–1155 (1975).

[b21] Bogaerts W. . Low-loss, low-cross-talk crossings for silicon-on-insulator nanophotonic waveguides. Optics letters 32, 2801–2803 (2007). 17909578 10.1364/ol.32.002801

[b22] Ma Y. . Ultralow loss single layer submicron silicon waveguide crossing for SOI optical interconnect. Optics express 21, 29374–29382 (2013). 24514491 10.1364/OE.21.029374

[b23] Morichetti F. . Non-invasive on-chip light observation by contactless waveguide conductivity monitoring. Selected Topics in Quantum Electronics, IEEE Journal of 20, 292–301 (2014).

[b24] Orlandi P. . Tunable silicon photonics directional coupler driven by a transverse temperature gradient. Optics letters 38, 863–865 (2013). 23503241 10.1364/OL.38.000863

[b25] Doerr C. R. . PDM-DQPSK silicon receiver with integrated monitor and minimum number of controls. Photonics Technology Letters, IEEE 24, 697–699 (2012).

[b26] Nakamura S. . Wavelength selective switching with one-chip silicon photonic circuit including 8 × 8 matrix switch. In Optical Fiber Communication Conference, OTuM2 (Optical Society of America, 2011).

