# Supplementary material for: 32 × 32 silicon electro-optic switch with built-in monitors and balanced-status units
Source: Sci Rep. 2017 Feb 9;7:42306. doi: 10.1038/srep42306 (PMC5299409; doi:10.1038/srep42306)

32 × 32 silicon electro-optic switch with built-in monitors and balanced-status units

Lei Qiao, Weijie Tang and Tao Chu*

Supplementary Table S1. The optimum working voltages and power consumptions for all units in the 32×32 switch in 'Cross' status.

Switch	Voltage (V)	Power (mW)	Switch	Voltage (V)	Power (mW)	Switch	Voltage (V)	Power (mW)	Switch	Voltage (V)	Power (mW)
S_{11}	0.87	0.17	S_{35}	9.98	17.96	S_{59}	0.95	1.05	S_{713}	0.87	0.26
S_{12}	1.01	2.12	S_{36}	0.92	0.64	S_{510}	0.99	1.68	S_{714}	0.95	1.24
S_{13}	0.96	1.25	S_{37}	1.05	3.31	S_{511}	0.89	0.36	S_{715}	0.92	0.74
S_{14}	1.02	2.24	S_{38}	0.91	0.55	S_{512}	0.92	0.64	S_{716}	1.04	3.85
S_{15}	0.99	1.68	S_{39}	1.04	2.91	S_{513}	0.93	0.84	S_{81}	0.89	0.27
S_{16}	1.16	5.34	S_{310}	0.93	0.84	S_{514}	0.97	1.55	S_{82}	0.99	1.98
S_{17}	0.95	0.95	S_{311}	0.92	0.74	S_{515}	0.86	0.17	S_{83}	0.98	1.67
S_{18}	0.92	0.55	S_{312}	0.93	0.84	S_{516}	0.96	1.44	S_{84}	0.98	1.57
S_{19}	1.05	2.52	S_{313}	0.97	1.55	S_{61}	0.88	0.29	S_{85}	0.94	0.94
S_{110}	1.13	4.75	S_{314}	0.86	0.17	S_{62}	0.94	1.03	S_{86}	1.00	2.00
S_{111}	0.95	0.95	S_{315}	0.96	1.44	S_{63}	0.91	0.73	S_{87}	0.99	1.78
S_{112}	0.82	0.08	S_{316}	1.00	2.60	S_{64}	1.05	3.68	S_{88}	0.93	0.74
S_{113}	1.00	1.80	S_{41}	0.86	0.17	S_{65}	0.93	0.84	S_{89}	0.95	1.05
S_{114}	1.04	3.02	S_{42}	1.02	2.96	S_{66}	1.16	7.08	S_{810}	0.97	1.36
S_{115}	0.92	0.64	S_{43}	0.94	1.03	S_{67}	0.95	0.95	S_{811}	0.97	1.46
S_{116}	1.04	3.22	S_{44}	0.97	1.55	S_{68}	0.93	0.74	S_{812}	0.99	1.88
S_{21}	0.83	0.08	S_{45}	0.97	1.46	S_{69}	0.95	1.05	S_{813}	0.92	0.64
S_{22}	0.99	2.08	S_{46}	1.04	3.02	S_{610}	1.05	3.05	S_{814}	0.98	1.76
S_{23}	0.98	1.76	S_{47}	0.99	1.83	S_{611}	1.02	2.65	S_{815}	0.98	1.76
S_{24}	0.95	1.14	S_{48}	0.9	0.45	S_{612}	0.86	0.26	S_{816}	0.97	1.65
S_{25}	0.94	0.94	S_{49}	0.99	1.78	S_{613}	0.96	1.44	S_{91}	0.89	0.27
S_{26}	1.05	3.26	S_{410}	0.97	1.46	S_{614}	0.99	2.18	S_{92}	0.97	1.36
S_{27}	0.99	1.78	S_{411}	1.00	2.10	S_{615}	0.93	0.93	S_{93}	1.02	2.24
S_{28}	0.95	1.05	S_{412}	0.91	0.55	S_{616}	0.95	1.33	S_{94}	1.01	2.02
S_{29}	0.96	1.15	S_{413}	0.86	0.17	S_{71}	0.85	0.17	S_{95}	1.02	2.14
S_{210}	1.01	2.22	S_{414}	0.88	0.26	S_{72}	1.03	3.30	S_{96}	1.06	2.97
S_{211}	0.97	1.46	S_{415}	0.96	1.44	S_{73}	0.97	1.65	S_{97}	1.11	3.89
S_{212}	0.89	0.36	S_{416}	0.96	1.44	S_{74}	1.01	2.53	S_{98}	1.03	2.06
S_{213}	0.91	0.55	S_{51}	0.84	0.08	S_{75}	0.94	0.94	S_{99}	1.06	2.54
S_{214}	1.02	2.75	S_{52}	1.05	3.89	S_{76}	0.98	1.67	S_{910}	0.95	0.86
S_{215}	1.01	2.53	S_{53}	0.96	1.44	S_{77}	0.97	1.36	S_{911}	0.92	0.55
S_{216}	0.92	0.74	S_{54}	1.00	2.20	S_{78}	0.98	1.57	S_{912}	1.04	2.60
S_{31}	0.78	0.08	S_{55}	0.96	1.34	S_{79}	0.96	1.25	S_{913}	0.99	1.68
S_{32}	0.97	1.65	S_{56}	1.07	3.96	S_{710}	0.94	0.94	S_{914}	1.01	2.02
S_{33}	0.98	1.86	S_{57}	1.10	4.29	S_{711}	0.98	1.76	S_{915}	1.05	3.26
S_{34}	1.02	2.75	S_{58}	0.85	0.17	S_{712}	0.92	0.74	S_{916}	0.91	0.55

Supplementary Table S2. The optimum working voltages and power consumptions for all units in the 32×32 switch in 'Bar' status.

Switch	Voltage (V)	Power (mW)	Switch	Voltage (V)	Power (mW)	Switch	Voltage (V)	Power (mW)	Switch	Voltage (V)	Power (mW)
S_{11}	1.15	7.02	S_{35}	1.02	2.75	S_{59}	1.11	4.44	S_{713}	1.15	7.25
S_{12}	1.04	2.91	S_{36}	1.12	5.60	S_{510}	1.06	3.18	S_{714}	1.03	3.19
S_{13}	1.08	4.10	S_{37}	0.98	1.67	S_{511}	1.15	6.79	S_{715}	1.07	4.71
S_{14}	1.03	2.47	S_{38}	1.15	6.21	S_{512}	1.09	4.91	S_{716}	0.93	0.93
S_{15}	1.06	3.18	S_{39}	0.99	1.78	S_{513}	1.09	5.01	S_{81}	1.17	7.25
S_{16}	0.93	0.65	S_{310}	1.10	4.84	S_{514}	1.03	3.09	S_{82}	1.01	2.53
S_{17}	1.11	4.22	S_{311}	1.09	4.69	S_{515}	1.13	7.01	S_{83}	1.04	3.22
S_{18}	1.17	5.27	S_{312}	1.08	4.54	S_{516}	1.02	3.06	S_{84}	1.04	3.02
S_{19}	1.04	2.29	S_{313}	1.02	2.75	S_{61}	1.10	6.27	S_{85}	1.10	4.73
S_{110}	0.95	0.95	S_{314}	1.14	7.30	S_{62}	1.05	3.99	S_{86}	1.03	2.68
S_{111}	1.13	4.52	S_{315}	1.03	3.40	S_{63}	1.07	4.49	S_{87}	1.04	2.91
S_{112}	1.27	9.78	S_{316}	0.98	1.96	S_{64}	0.96	1.34	S_{88}	1.09	4.03
S_{113}	1.07	3.42	S_{41}	1.12	7.06	S_{65}	1.09	4.80	S_{89}	1.05	3.05
S_{114}	1.01	2.22	S_{42}	0.98	1.86	S_{66}	0.87	0.26	S_{810}	1.06	3.39
S_{115}	1.14	5.70	S_{43}	1.06	4.13	S_{67}	1.11	4.44	S_{811}	1.05	3.15
S_{116}	0.98	1.76	S_{44}	1.03	3.09	S_{68}	1.14	5.24	S_{812}	1.02	2.55
S_{21}	1.16	8.58	S_{45}	1.04	3.22	S_{69}	1.09	3.82	S_{813}	1.11	5.33
S_{22}	1.01	2.63	S_{46}	0.98	1.67	S_{610}	0.99	1.68	S_{814}	1.01	2.42
S_{23}	1.03	3.09	S_{47}	1.03	2.78	S_{611}	0.99	1.98	S_{815}	1.02	2.86
S_{24}	1.07	4.17	S_{48}	1.15	6.21	S_{612}	1.14	6.61	S_{816}	1.01	2.63
S_{25}	1.09	4.69	S_{49}	1.06	3.39	S_{613}	1.04	3.43	S_{91}	1.17	7.25
S_{26}	0.98	1.67	S_{410}	1.06	3.39	S_{614}	0.99	2.18	S_{92}	1.08	4.00
S_{27}	1.03	2.68	S_{411}	1.01	2.22	S_{615}	1.06	4.35	S_{93}	1.04	2.70
S_{28}	1.09	4.14	S_{412}	1.12	5.82	S_{616}	1.03	3.50	S_{94}	1.05	2.84
S_{29}	1.07	3.53	S_{413}	1.17	8.07	S_{71}	1.14	7.87	S_{95}	1.04	1.56
S_{210}	1.01	2.22	S_{414}	1.14	7.07	S_{72}	0.97	1.65	S_{96}	1.00	1.71
S_{211}	1.06	3.50	S_{415}	1.04	3.54	S_{73}	1.03	3.19	S_{97}	0.97	1.16
S_{212}	1.16	7.08	S_{416}	1.02	3.06	S_{74}	0.99	1.98	S_{98}	1.05	2.52
S_{213}	1.12	5.82	S_{51}	1.14	7.98	S_{75}	1.07	4.07	S_{99}	1.02	1.84
S_{214}	1.00	2.00	S_{52}	0.94	1.03	S_{76}	1.04	3.12	S_{910}	1.14	4.45
S_{215}	1.00	2.30	S_{53}	1.03	3.19	S_{77}	1.06	3.50	S_{911}	1.13	4.63
S_{216}	1.08	4.97	S_{54}	1.01	2.53	S_{78}	1.05	3.15	S_{912}	1.00	1.80
S_{31}	1.16	9.16	S_{55}	1.05	3.57	S_{79}	1.06	3.50	S_{913}	1.05	2.84
S_{32}	1.03	3.30	S_{56}	0.95	1.14	S_{710}	1.07	3.85	S_{914}	1.03	2.27
S_{33}	1.01	2.63	S_{57}	0.95	0.95	S_{711}	1.02	2.65	S_{915}	0.97	1.46
S_{34}	0.99	1.98	S_{58}	1.23	8.12	S_{712}	1.09	4.80	S_{916}	1.11	5.11

Supplementary Figure S1. Transmission spectra of all output ports in the 32×32 switch when lights input all ports and all switch units were in ‘Cross’ status.

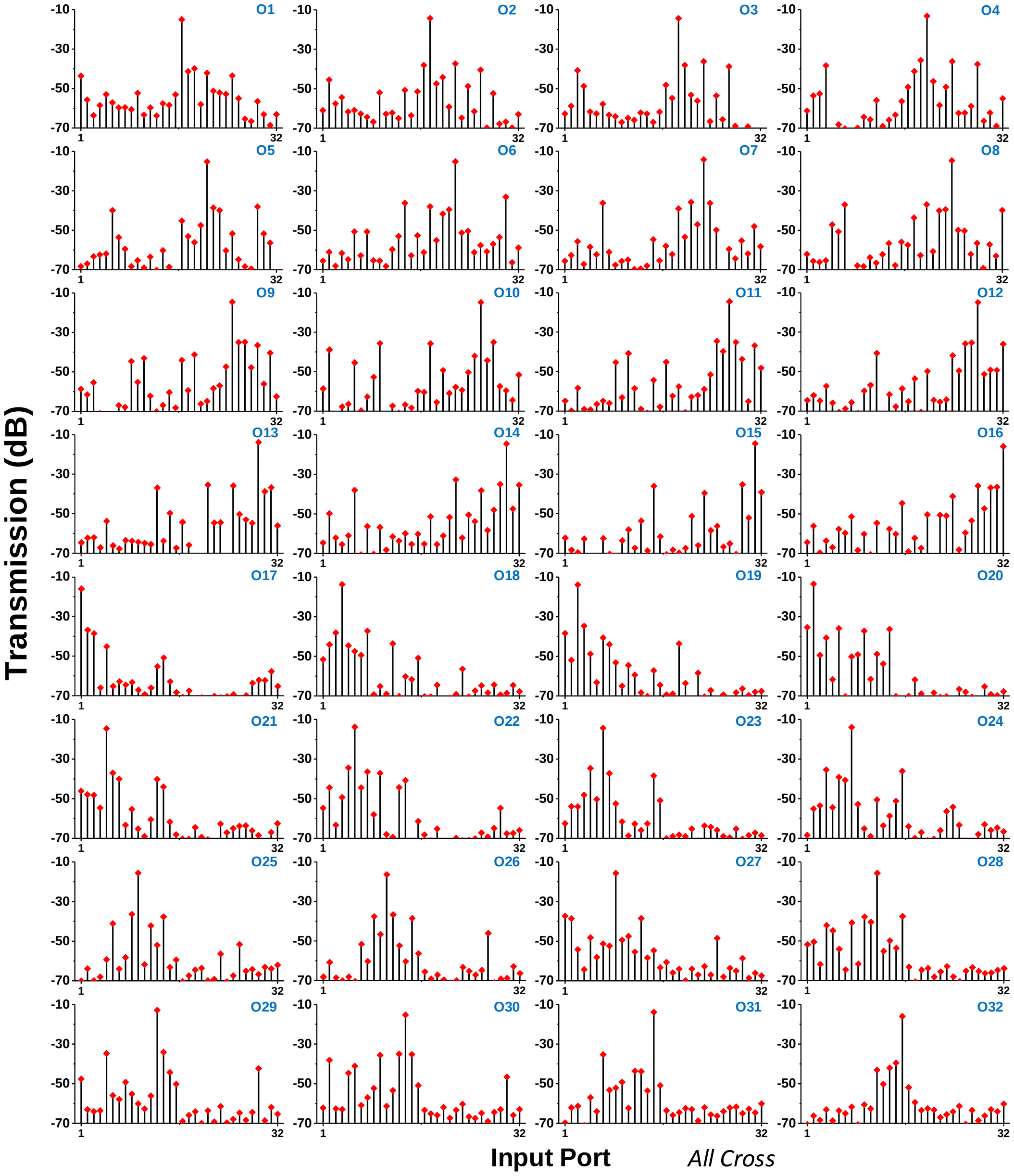

Supplementary Figure S2. Transmission spectra of all output ports in the 32×32 switch when lights input all ports and all switch units were in 'Bar' status.

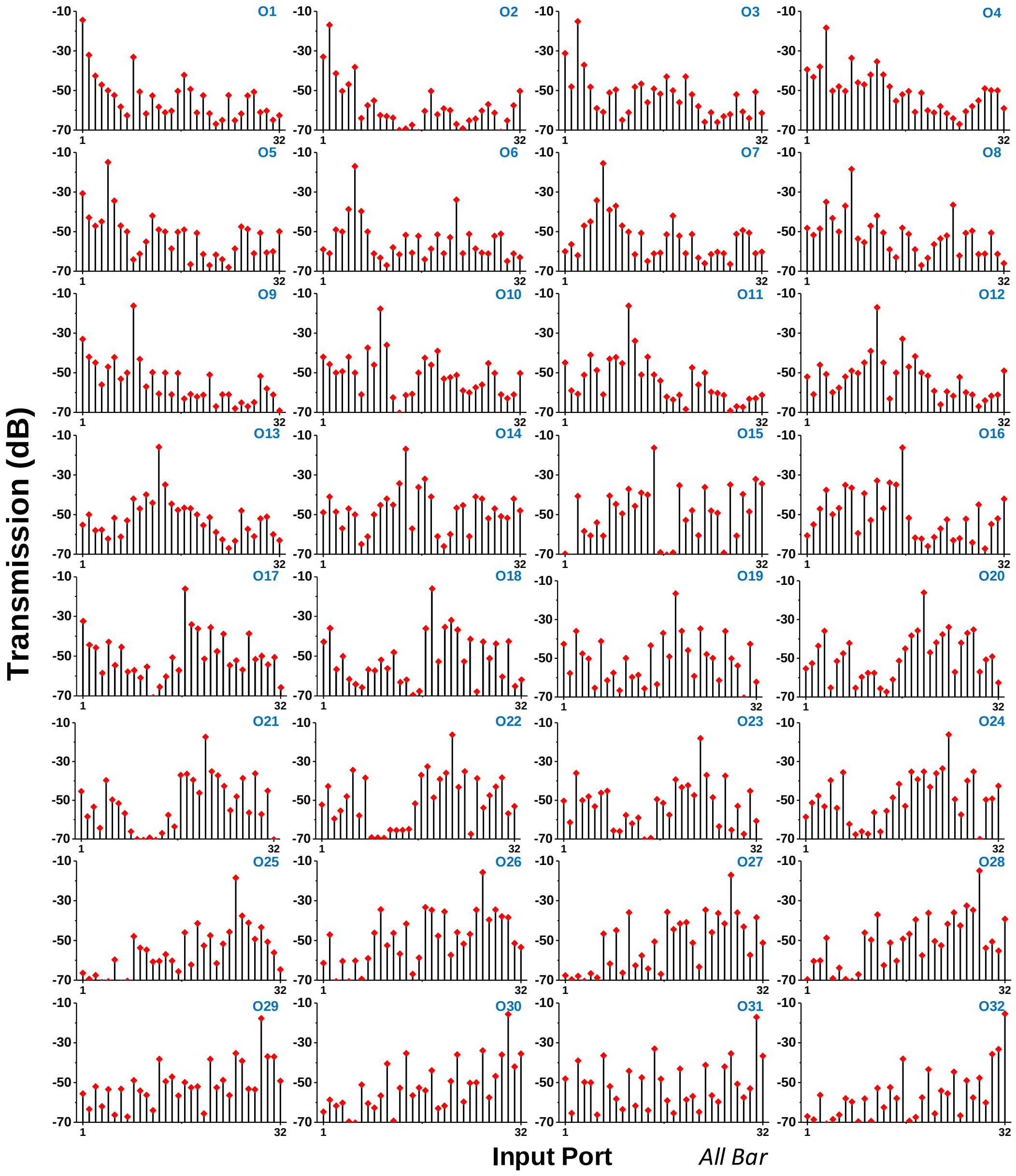

Supplement: Supplementary Information [file srep42306-s1.pdf]
